# Development of synthetic selfish elements based on modular nucleases in *Drosophila melanogaster*

**DOI:** 10.1093/nar/gku387

**Published:** 2014-05-06

**Authors:** Alekos Simoni, Carla Siniscalchi, Yuk-Sang Chan, David S. Huen, Steven Russell, Nikolai Windbichler, Andrea Crisanti

**Affiliations:** 1 Department of Life Sciences, Imperial College London, South Kensington Campus, London SW7 2AZ, UK; 2 Department of Genetics, University of Cambridge, Downing Street, Cambridge CB2 3EH, UK; 3 Centre of FunctionalGenomics, University of Perugia, S. Andrea delle Fratte, 06132, Perugia, Italy

## Abstract

Selfish genes are DNA elements that increase their rate of genetic transmission at the expense of other genes in the genome and can therefore quickly spread within a population. It has been suggested that selfish elements could be exploited to modify the genome of entire populations for medical and ecological applications. Here we report that transcription activator-like effector nuclease (TALEN) and zinc finger nuclease (ZFN) can be engineered into site-specific synthetic selfish elements (SSEs) and demonstrate their transmission of up to 70% in the *Drosophila* germline. We show here that SSEs can spread via DNA break-induced homologous recombination, a process known as ‘homing’ similar to that observed for homing endonuclease genes (HEGs), despite their fundamentally different modes of DNA binding and cleavage. We observed that TALEN and ZFN have a reduced capability of secondary homing compared to HEG as their repetitive structure had a negative effect on their genetic stability. The modular architecture of ZFNs and TALENs allows for the rapid design of novel SSEs against specific genomic sequences making them potentially suitable for the genetic engineering of wild-type populations of animals and plants, in applications such as gene replacement or population suppression of pest species.

## INTRODUCTION

**Figure 1.**
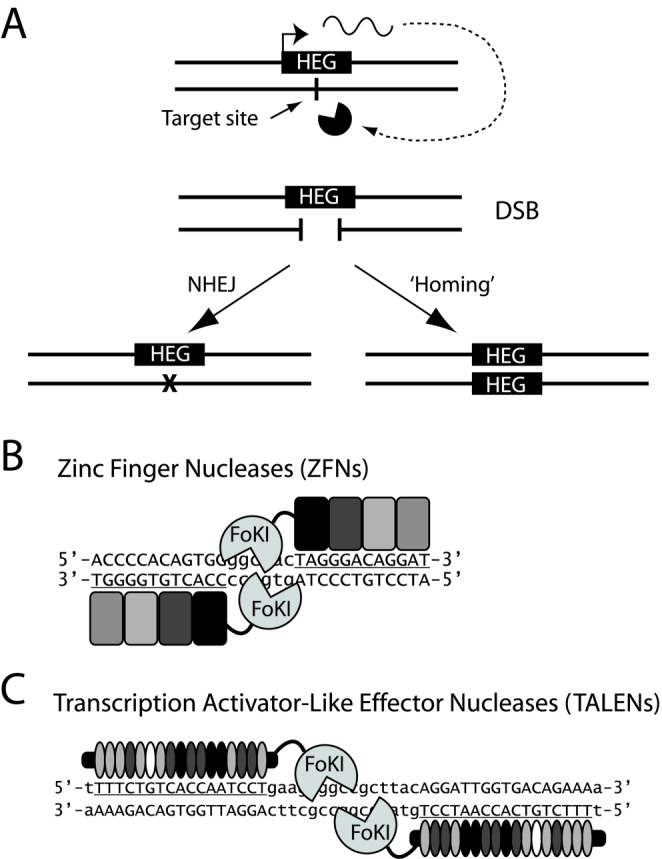
Model of HEG-mediated homing and of ZFNs and TALENs. (**A**) Mode of action of HEGs. When a HEG is expressed in a *trans*-heterozygous cell (top), it recognizes its target site on the homologous chromosome generating a DSB (middle). The broken chromosome activates the repair machinery of the cell that can employ the non-homologous end-joining pathway (bottom left) or homologous recombination (bottom right), using the HEG-containing chromosome as a repair template. A HEG heterozygous is converted into a HEG homozygous, a process called ‘homing’. (**B**) Schematic of ZFNs and (**C**) TALENs. They both act as a dimer in which each monomer binds on complementary DNA strands (binding sequences are underlined and in capital letter) and the FoKI nuclease directs its activity to the spacer between the recognition sequences generating a 5’-overhang. Each ZFN module recognizes a DNA triplet and four fingers are linked together to selectively recognize a 12 bp target sequence. Individual TALEN modules bind to single nucleotide and 17 modules are assembled together to form a functional nuclease.

Selfish genes are DNA elements that have evolved to enhance their own transmission relative to the rest of the genome. Population genetics and genome sequence analysis indicates that these elements have played a key role in the evolution of host genomes in a wide range of organisms (1). Naturally occurring selfish elements include transposable elements, meiotic drive chromosomes, sex ratio distorting elements and homing endonuclease genes (HEGs). HEGs are highly specific endonucleases that generate double-strand breaks (DSB) at specific loci in the host genome (2). The recognition sequence is usually 14–40 bp long and occurs generally only once in the host haploid genome. As schematized in Figure 1A, the coding sequences of HEGs are located in the middle of their own recognition sequence at the same locus on the homologous chromosome, thus preventing the HEG-bearing chromosome from being cleaved. Once expressed, HEGs cleave the target sequence and the broken chromosome can activate the recombinational repair machinery of the cell, which then uses the homologous HEG-containing allele as a corrective template. As a consequence, a HEG heterozygote can be converted into a homozygote, a process known as ‘homing’ (Figure 1A). In metazoans, if homing occurs in the germ cells, HEGs are transmitted to the progeny at a frequency higher than the expected Mendelian ratio, and can therefore spread within a host population.

It has been proposed that HEGs which have been engineered to recognize selected target sequences could be exploited to generate drive systems for genetic control of disease vectors (3,4) as well as a range of other medical and ecological applications (5). This can be achieved by the replacement of wild population with less harmful variants, for instance insects that do not transmit the disease, or by population suppression of the disease vector in a specific area. However, engineering HEG nucleases to target custom sequences without impairing catalytic activity has proven more complex than initially anticipated (6).

We reasoned if the ‘selfishness’ of HEGs, the ability to invade host genomes, is exclusively linked to the sequence specificity of the endonuclease and to the genomic location of their recognition site. Do HEGs possess additional biological properties or activities? To answer this question, we investigated whether synthetic, modular nucleases could be transformed into synthetic selfish elements (SSEs) using the functional principles of the HEG system as a model.

Transcription activator-like effector nuclease (TALEN) and zinc finger nuclease (ZFN) are two platforms that have recently arisen as powerful tools for genome editing in a highly site-specific manner (reviewed in (7)). Unlike HEGs, the DNA binding domain of both TALENs and ZFNs consists of modular domains that can readily be rearranged to target virtually any specific DNA sequences (Figure 1B and C). When linked to an independent FoKI DNA nuclease domain (8), the reprogrammable DNA binding domains of TALENs and ZFNs direct the nuclease activity to unique DNA sites, generating a DSB with a 5’ overhang. TALENs and ZFNs both function as dimers, in which each monomer binds to sequences on complementary strands of the DNA, and the catalytic activity of the FoKI is directed to the spacer between the two recognition sequences (Figure 1B and C). The high sequence specificity of both TALENs and ZFNs is conferred by the long recognition sequence (each monomer recognizes 12–20 bp) and further specificity can be achieved by employing two FoKI variants which are active only as a heterodimer, therefore reducing the possibility of homodimers cutting at off-target sites (9).

In this study, we utilized two well-characterized and highly active endonucleases, *ZFN-AAVS1* and *PPP1R12C* TALEN-R, a ZFN and a TALEN respectively, each targeting a unique sequence in the human PPP1R12C gene, associated with the AAVS1 locus (10,11). We investigated whether *ZFN-AAVS1* and *PPP1R12C* TALEN-R could be converted into SSEs in susceptible *Drosophila* strains engineered to carry the corresponding target sequences.

The ability of HEG-like selfish elements to serve as gene drive systems and spread into a target population relies on the expression and activity of the nucleases in the gametes. For this purpose, we expressed a number of SSEs under control of a spermatogenesis promoter from the *Rcd-1r* gene (12,13). To facilitate the read out of the system, we developed a reporter system based on a combination of fluorescent and phenotypic markers similar to our previously described model to characterize HEG activity in *Drosophila* (13,14).

Here we describe the activity of our novel SSEs in the male germline, with homing to the target sites at frequencies of 49% and 34% for TALEN- and ZFN-based SSE versions, respectively. We found that many of these homing events (40% for TALENs and 75% for ZFNs) generated target chromosomes capable of further rounds of homing in subsequent generations.

## MATERIALS AND METHODS

### Generation of constructs and transgenics

The donor constructs are a derivate of our previously described pDarkLime vector (13,14), where the *I-Sce*I ORF has been replaced by the TALEN or ZFN sequences (see below). The donor cassette was cloned in a *Not*I site which destroys the nuclease target site within the eGFP ORF. This vector contains the *Rcd-1r* promoter, ß-56D 3’-UTR, a promoterless red fluorescent protein (RFP) and SV40 polyA site as described in (13). In addition it contains a functional *mini-white* gene and an *att*B site for ϕC31 site-specific integration.

The PPP1R12C-TALEN was provided by Sangamo BioScience Inc. (accession number 101079) (15) and was cloned by polymerase chain reaction (PCR) with the primers GTATGGGAGACCTCATGGTGGACTTGAGGACAC and TGATCAGCGGGTTTAAACTG into the *Bsa*I and *Xba*I site in pDarkLime.

ZFN-AAVS1 was provided by Sangamo BioScience Inc. (10). The left and right ZFN-AAVS1 were cloned into a *Xba*I-*Mlu*I and *Age*I-*Bfr*I sites, respectively, introduced by PCR together with a self-cleavage F2A stuttering signal between (16), using the following primers: AGGCTGATCAGCGGGTTTAAACGG; TACATTACGCGTATGGACTACAAAGACCATGA; TATATTACCGGTAGATCTGAAGTTGATCTCGC and TAATTCTTAAGATGGCTGAGAGGCCCTTCCAG.

The target constructs are a derivate of our wDarkLime target construct (14) in which the TALEN and ZFN target sites (see Figure 1B) were inserted in-frame with the 3xP3-eGFP via the unique *I-Sce*I site.

The ZFN-AAVS1-Long was generated by insert 4 kb of genomic DNA from Canton-S wild-type strain upstream of the *Rcd-1r* gene promoter via an *Nhe*I site using the primers AATATTGCTAGCCATACGTGTTTGTGAGC and TATACTAGTGCGGCCGCTCCAAAATCCCGTTACAGC.

**Figure 2.**
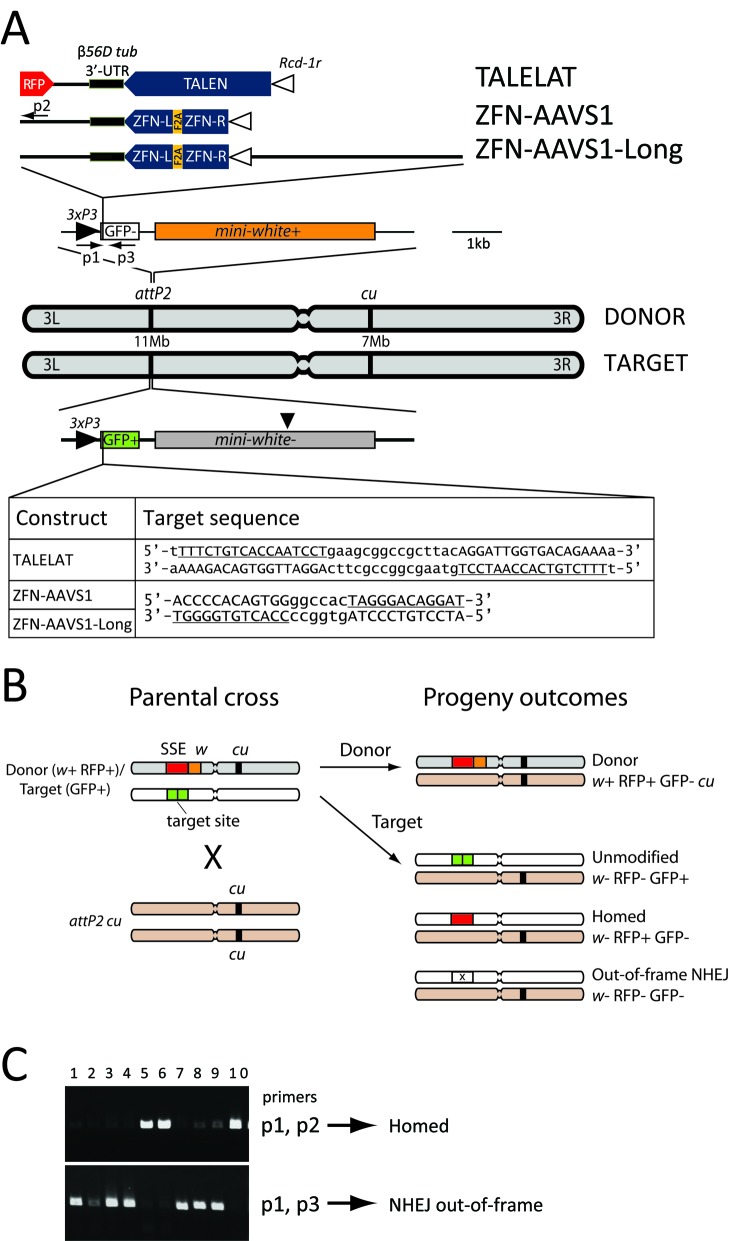
Schematic representation of the genetic markers used to follow the structure of the progeny. (**A**) Donor and target constructs were inserted by ΦC31site-specific integration in the same *attP2* docking line (on chromosome *3L*, position 11 063 638 bp). Donor nuclease sequences are inserted as cassette (top) within their corresponding target site interrupting the green fluorescent protein (GFP) coding sequence. Three different SSEs were constructed encompassing the following elements: the male germline promoter *Rcd-1r* (white triangle), a TALEN or a ZFN-pair nuclease (blue shape) and the *ß56D-tubulin* 3’-UTR (black bar). The TALELAT construct carries a RFP marker gene driven by the eye-specific promoter 3xP3 (black triangle). The left and the right ZFNs are separated by a Furin-2A self-cleavage ribosomal stuttering peptide (yellow). The donor constructs are adjacent to a functional *mini-white* gene (orange box) that restores the red pigmentation in the fly's eyes as phenotypical marker and the recessive marker *curled* (*cu*), on chromosome *3R* (position 7 023 314). The recipient (target) chromosome carries the nuclease target sequence (as shown. The nuclease recognition sequences are underlined and in capital letters) in-frame with a functional GFP gene (green box), driven by the eye-specific promoter 3xP3. The *mini-white* marker in the target line was inactivated by a frame-shift mutation (marked by an arrow head). (**B**) Schematic of the homing assay. Donor/target *trans*-heterozygous flies are crossed to attP2 *cu* flies (the genetic background). When the nucleases are expressed in the germline, the target site is cleaved and the chromosome repair mechanisms can lead to different progeny outcomes, which can be discriminated by fluorescent and phenotypical markers, as indicated. Donor and target chromosome (marked in grey and white, respectively) can be discriminated by the presence of dominant *mini-white* (*w*) and recessive *curled* (*cu*) markers. We defined ‘homing’ as any recombination event which leads to the conversion of the target chromosome into a nuclease expressing chromosome. (**C**) PCR reactions were performed on *w-* GFP-negative flies with different sets of primers to distinguish HR-dependent repair events from imprecise NHEJ. The combination P1–P2 generates a PCR amplicon only in the case of homing of the locus while the combination P1–P3 is diagnostic for NHEJ events. Lanes 1–10 were loaded with PCR reactions generated from GFP-negative flies derived from TH males crossed to wt females.

Transgenic fly lines were produced by ϕC31 integrase-mediated insertion into the *attP2* docking line (3L: 11 063 638). The TALELAT donor and AAVS1 target were inserted into an otherwise unmarked chromosome. ZFN-AAVS1, ZFN-AAVS1-Long donor and TALELAT target were inserted into an *attP2* chromosome marked with *curled* (*cu*). The schematic of the homing assay and the possible progeny outcomes from the *trans*-heterozygous cross depicted in Figure 2B and described in the text in reference to the *curled* marker exemplifies the situation of the TALELAT line. For ZFN-AAVS1 and ZFN-AAVS1-Long, the *curled* marker is on the target chromosome, and the progeny was classified accordingly.

All injections were performed at the Fly Facility, Department of Genetics, University of Cambridge, UK.

### Homing assay

Donor and target lines were crossed to generate *trans*-heterozygous flies. The progeny were crossed in mass matings as follows:

*y w*;; attP2[ZFN-AAVS1 Donor]/attP2 [ZFN-AAVS1 target] *cu* × *y w*;;attP2 *cu*/attP2 *cu*

*y w*;; attP2[ZFN-AAVS1-Long Donor]/attP2 [ZFN-AAVS1 target] *cu* × *y w*;;attP2 *cu*/attP2 *cu*

*y w*;; attP2[TALELAT Donor] *cu*/attP2 [TALELAT target] × *y w*;;attP2 *cu*/attP2 *cu*

*y w*;;attP2[target]/attP2 *cu* progeny were identified by the lack of eye pigmentation (*mini-white-*) and analysed for the loss of GFP, switch from GFP to RFP expression or molecular characterization by PCR.

The number of total chromosome screened is indicated in Table 1. Analysis of functional F2 products was carried out as following:

**Table 1.** Activity of the SSEs compared to a natural HEG in our *Drosophila* homing assay

	Male TH x wt			Female TH x wt			
Line	GFP loss (counts)	Homing fraction (counts)	Fraction of targets homed	GFP loss (counts)	Homing fraction (counts)	Fraction of targets homed	Functional homing
TALELAT	70.2% (998/1422)	69.8% (697/998)	49%	1.4% (21/1487)	81% (17/21)	1.1%	40% (15/36)
ZFN-AAVS1	86.1% (366/425)	39.5% (117/296)	34%	21.3% (110/517)	nd	nd	75% (15/20)
ZFN-AAVS1- Long	85.5% (483/565)	39.7% (173/435)	34%	11.9% (119/997)	nd	nd	nd
*I-Sce*I*	37.2% (1273/3422)	61.4% (782/1273)	23%				100%

The percentage (and counts) of GFP loss, homing fraction and total fraction of targets homed is shown. The values refer to the white-eye flies progeny (target chromosome) according to the phenotypical markers described in Figure 2B. The GFP loss is likely to be an underestimation of the nuclease activity since it does not take into account NHEJ repairs that restore a functional GFP as well as HR with the sister chromatid. ‘Functional homing’ indicates the fraction of homing events that lead to a SSE able to perform a second round of homing in the next generation (calculated as the fraction of F2 crosses in which the loss of GFP was observed). *Data on *I-Sce*I were originally described and published in (13). nd: not done.

*y w*;; attP2[Donor]/attP2 [target] *cu* × *y w*;;attP2 [target]/attP2 [target] from which the targeted progeny *y w*;;attP2[targeted]/attP2 [target] flies (RFP^+^, white eye) were individually backcrossed to fresh y w;;attP2 [target] flies and the progeny screened to identify deviation of the expected 1:1 ratio of GFP^+/−^ positive and negative as an indication of the Donor nuclease activity. Fluorescent markers were analysed on a Nikon inverted microscope (Eclipse TE200) to detect GFP and RFP expression.

The frequency of cleavage, homing and non-homologous end joining (NHEJ) rates were calculated as previously described (6,13,14). Briefly, cleavage rate was calculated as the fraction of the target (*w-*) chromosome with no GFP expression. This does not take into account altogether the fraction of perfect (in-frame) NHEJ repairs and homologous recombination (HR) with the sister chromatid (indistinguishable from uncut chromosomes). Homing rate was calculated as the fraction of *w-* progeny gaining RFP expression (for TALELAT SSEs) or by PCR assay (see below), for ZFN SSEs. The total fraction of homed flies is presented as the fraction of homing events on the cleaved chromosomes.

The fraction of successful F2 homing events was calculated by scoring the statistically significant deviation (Chi-square, *P* < 0.05) from the expected 1:1 fraction of GFP^+^:GFP^−^ in the progeny of F1 homed fly (*w^−^* RFP^+)^ individually crossed to wild-type flies.

### PCR analysis

DNA was prepared by maceration of flies in a 10 mM TrisCl (pH8.2), 1 mM EDTA, 25 mM NaCl and 200 μg/ml Proteinase K buffer. The position of the primers is shown in Figure 2. GFP-negative flies from F1 progeny of ZFN-AAVS1 and ZFN-AAVS1-Long were screened by PCR using primers p1: ATAGAGGCGCTTCGTCTACG; Primer p2: CGCGCAGCTTCACCTTGTAG and primer p3:TCGTCCTTGAAGAAGATGGTG. When primers p1 and p2 gave a positive product, the target chromosome was scored as homed. A number of GFP-negative flies were PCR amplified with primers p1 and p3 and sequenced to characterize out-of-frame NHEJ events (Figure 2 and 5). Analysis of dysfunctional F2 products was performed with the primers (i) ATAGAGGCGCTTCGTCTACG, (ii) CGCGCAGCTTCACCTTGTAG, (iii) GATCGAGAATTCGATTGATTTCCG and (iv) TCCTCGGCTCTGGCCACATT.

### Off-target effect assay

Online tools to assess genome-wide off-target effects for ZFN (17) and TALEN (18) do not predict any putative off-target sites for the nucleases used in this study in the *Drosophila* genome. To identify genomic sequences closely resembling the nuclease target sites, we searched the *Drosophila* genome sequences via the Blast tool Ensemble (http://www.ensembl.org/Multi/blastview) with the following parameters: no optimisation for search sensitivity; no filter; no RepeatMasker; 4 as word size for seeding alignments and no gaps allowed. The resulting sequences were sorted by Raw alignment score and alignment length. We allowed a spacer length between the recognition sequences of 5 and 6 bp for ZFN and 15 to 25 bp for TALEN. The top score sequences were PCR amplified from genomic DNA of eight SSE expressing flies after at least 10 generations of siblings interbreeding and sequenced.

### Oviposition and hatching rate experiments

Two to three females were allowed to mate and lay eggs on standard cornmeal food for 3 consecutive days; the total number of eggs laid per female per day was counted. The total number of eclosing adults was counted. At least five replicates per genotype were tested and the average with standard error of the mean (SEM) displayed. One-way ANOVA with Tukey's post-test between genotypes was performed in GraphPad Prism4 Software.

### Population experiments

TALELAT *trans*-heterozygous (donor/target) males were crossed to target females to eliminate the *w^+^* allele from the donor chromosome. Twenty-five *w-*, RFP^+^ progeny were selected and introduced into populations of 50 males and 50 females homozygous target flies. After mating, the adults were discarded and the progeny analysed for the presence of the fluorescent markers: 100 (+/−10) randomly selected flies were used to establish the next generation.

Population experiments for the ZFN SSE were established as follows: Population 1, 2 and 3: 50 *trans*-heterozygous (donor/target) males were crossed to 75 target virgin females and 75 target males; Population 4: 40 *trans*-heterozygous (donor/target) males were crossed to 90 virgin target females and 80 males; Population 5: 30 *trans*-heterozygous (donor/target) males crossed to 100 target virgin females and 100 target males. After mating, the adults were discarded, the progeny scored for the presence of the GFP marker and 400 (+/−20%) flies used to establish the next generation.

As a control, we established a refractory (non-cleavable) target line initially obtained from a GFP-negative individual carrying deletions and insertions on the target site from an imprecise NHEJ event, in which nuclease recognition target site had been destroyed. We introduced 25 *trans*-heterozygous (donor/target) males into population of 50 males and 50 females homozygous refractory target flies. After mating the adults were discarded and the progeny analysed for the presence of the RFP expression (for the TALELAT line) or by PCR assay (for the ZFN line).

### Stochastic simulation

The dynamics of the population simulations are described by a stochastic random mating model of discrete non-overlapping generations. The model considers the following parameters: the initial number of males and females (50 males and 50 females for TALEN data and 200 males and 200 females for ZFN-AAVS1 data), the number of eggs per female (40), population size (100 for TALEN and 400 for ZFN-AAVS1) and release size (number of *trans*-heterozygous donor flies introduced: 25 for TALEN and 100 for ZFN-AAVS1). The model assumes four genotypes with no fitness difference among them: wild-type (i.e. target sites, T), donor (functional SSE, D), non-functional donor (D^N^) and out-of-frame NHEJ (i.e. mutation in the target site, N). The model randomly performs crosses of males and females of the available genotypes according to the frequency of each and capping the population at the specified size.

Populations start as a mixture of male D/T trans-heterozygotes and male and female T/T homozygotes. In male D/T trans-heterozygotes, cleavage of the target chromosomes followed by homing occurs and four classes of alleles are generated: D (donor plus homed target), T (unmodified target), D^N^ (non-functional homed) and N (products of misrepair). The frequencies at which the alleles are generated are based on experimental data as reported in Table 1. D, D^N^ and N are resistant to further cleavage and D^N^ is not able to home. All other crosses produce gametes in Mendelian proportions. All genotypes have equal survival and fertility and each female mate with a single male chosen randomly. The model returns the allelic frequency for each genotype in the population for each generation. The model has been written in C# and simulations generated in Microsoft Visual Studio 2010.

## RESULTS

### Construction of SSEs

Homing requires the nuclease donor to be situated at the same chromosomal location to the target site on the homologous chromosome. To achieve this we performed site-specific integration of donor and target constructs into the same docking site on chromosome 3L (attP2) with the ϕC31 integrase system (19) (Figure 2A). We designed a reporter system in *Drosophila* to monitor the ability of *ZFN-AAVS1* (11) and *PPP1R12C* TALEN-R (15) to invade a target sequence by homing. The reporter system is based on a set of constructs which carry the nuclease target sequence inserted in-frame within a functional eGFP marker gene regulated via an eye-specific promoter (designated as 3xP3) (20) linked to an inactive *mini-white* gene (Figure 2A).

We have generated three SSE donor constructs in which *ZFN-AAVS1* and TALEN were placed under the control of a spermatogenesis-specific promoter from the *Rcd-1r* gene and the *ß-Tub56D* 3’-UTR, a combination previously described to yield spermatogonial expression and homing in a *Drosophila* HEG assay (6,13). The donor constructs include a functional *mini-white* gene, which restores red pigmentation to the eye, to distinguish chromosomes containing the donor (*mini-white^+^*) from those carrying the target construct (*mini-white^−^*). In the donor construct, the GFP target sequence is disrupted by the nuclease expressing cassette that renders the donor chromosome refractory to the action of the nucleases (Figure 2A).

The TALELAT construct contains both the 3xP3 RFP, as a reporter, and the *Rcd-1r PPP1R12C* TALEN-R transcription unit. The latter one encodes a TALEN monomer that acts as a homodimer to recognize a palindromic site of two 17 bp sequences separated by a 15 bp spacer (Figure 2A).

The ZFN-AAVS1 construct encodes two ZFN proteins, each fused to the FoKI nuclease domain, in an obligate heterodimer configuration (9). The ZFNs recognize two sets of 12 bp sites separated by a 6-bp spacer (Figure 2A). The left and right ZFN coding sequences were separated by the self-cleaving F2A peptide (21) to allow the transcription of the two protein genes as a single mRNA. A third construct, ZFN-AAVS1-Long, resembles ZFN-AAVS1 except for the addition of an extra 4-kb of non-coding DNA upstream of the *Rcd-1r* promoter (Figure 2A) in order to assess whether the size of the donor cassette affected its targeting activity.

### Activity of TALEN- and ZFN-based SSE *in vivo*

**Figure 3.**
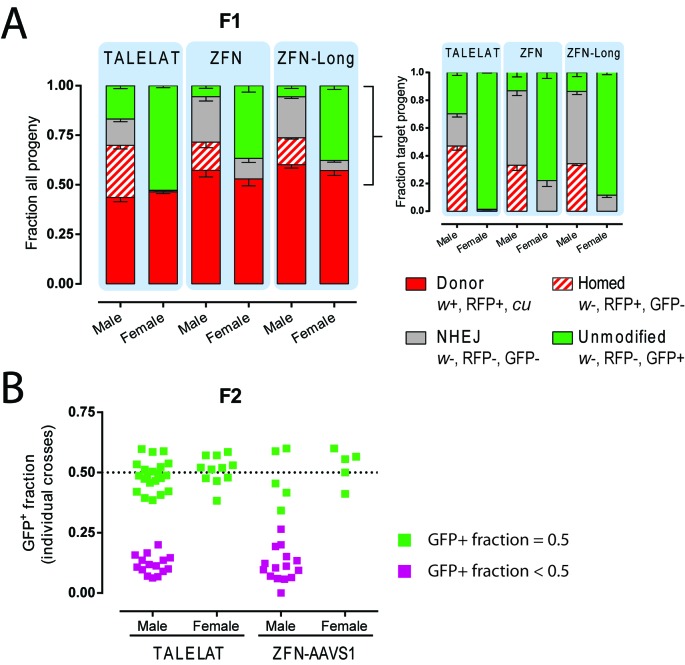
Phenotypical analysis of SSEs activity *in vivo*. (**A**) Phenotypic analysis of progeny originating from crosses of donor/target *trans*-heterozygous (TH) and wild-type flies according to the markers described in Figure 2. ‘Male’ and ‘female’ denote the gender of the *trans*-heterozygous parent. The bars indicate the fraction of offspring carrying the donor chromosome (red; this is directly inherited from parent to offspring and is not the result of nuclease activity), homed target chromosome (striped red; fraction of homing), unmodified target (green) and out-of-frame NHEJ (grey). The inset on the right shows the phenotypic analysis of progeny from target-chromosome only (*w-*) as reported in Table 1. Error bars indicate SEM between independent crosses. (**B**) Phenotypic analysis of GFP expression in F2 progeny from F1 homed flies individually crossed to fresh target flies. Each dot indicates the GFP fraction of an individual cross outcome. The crosses in which the GFP-positive fraction statistically differs from 0.5 (Chi-square, *P* < 0.05) are shown in purple.

We crossed the donor and target lines to generate *trans*-heterozygous progeny in which the SSE donor constructs were paired with their corresponding cleavage sites at the same chromosomal locus (Figure 2B). We then crossed male *trans*-heterozygotes to *attP*2 *cu* female flies, the genetic background of all the stocks used in this study and henceforth referred to as wild-type (wt). In *Drosophila* males meiosis occurs in the absence of recombination (22) and thus the use of dominant (*mini-white*) and recessive (*curled*, *cu*) markers allows the precise tracking of donor and target chromosomes in the progeny (Figure 2B). Repair events induced by the cleavage activity of the nuclease can result in different phenotypes in the progeny, which can be identified by the presence of distinct fluorescent markers (Figure 2B). Cleavage of the target site was measured by scoring the loss of GFP expression in the white-eye progeny, which had inherited the target chromosome (Table 1 and Figure 3A). However, if the broken chromosome is perfectly repaired, either by HR with the sister chromatid (14) or by in-frame NHEJ, this would be indistinguishable from the original target chromosome, and it was scored as ‘unmodified’. The total number of cleavage events (as reported in Table 1) is therefore likely to be underestimated in this assay. To distinguish HR from imprecise NHEJ events, both of which lead to the loss of GFP, we used either a fluorescence marker (switch from GFP to RFP expression in the white-eye F1 offspring) when examining the TALEN crosses or a PCR assay for ZFN-induced cleavage (Figure 2C).

Recombination events associated with DSB repair can involve different mechanisms. HR with cross-over or no cross-over of the flanking regions could lead to co-conversion of the markers flanking the DSB site. The presence of the recessive *cu* marker on the donor chromosome allows us to distinguish homing events (in which the donor cassette is copied to the homologous chromosome: RFP^+^ in a *w-* and *cu*^−^ background) from co-conversion of the *mini-white* gene (if the co-conversion tract is bigger than 3.9 kb—i.e. the distance between the DSB site and the mutation that inactivate the *mini-white* gene in the target chromosome). The outcomes of the latter events (RFP^+^ in a *w^+^* and *cu^−^* background) are distinguished from progeny in which the donor chromosomes are vertically transmitted, which are RFP^+^ in *w*^+^ and *cu* background (Figure 2B). Co-conversion of the *mini-white* gene was not observed in any of the progeny from male *trans*-heterozygotes, and although we lack an equivalent set of markers on the left side of the target site (which limits our ability to distinguish the precise mechanism of recombinational repair) we concluded that our assay allowed us to detect all relevant outcomes shown in Figure 2B. Thus, for the scope of this study, any recombination events that lead to the conversion of a target chromosome into a donor carrying chromosome were classified as ‘homed’ (Figures 2B, 3A and Table 1).

We also crossed TALELAT donor/ZFN-AAVS1 target *trans*-heterozygous males to wild-type female flies as a control. Since the ZFN-AAVS1 site is refractory to the TALELAT activity, the expected outcome of this cross is 1:1 ratio of *w-* GFP^+^ to *w*^+^ RFP^+^ (i.e. donor and target chromosomes are inherited to the progeny without rearrangements).

The analysis of different crosses revealed that both nucleases were highly active *in vivo* (Table 1 and Figure 3A), with cleavage activity at the target site (GFP loss) ranging from 70% to 86% of chromosomes analysed. The TALELAT successfully invaded the recipient site via homing in 69.8% of the cleaved chromosomes (gaining expression of the RFP marker in *w*^−^ flies), corresponding to 49% of the available loci (in Figure 3A). PCR-based analysis of the cleaved chromosomes after ZFN-AAVS1 and ZFN-AAVS1-Long activity showed that 39.5% and 39.7%, respectively, of the cleaved sites were repaired via HR, generating SSE invasion (i.e. homing) in 34% of total target chromosomes in the offspring. By comparison, in the TALELAT donor/ZFN-AAVS1 target *trans*-heterozygous control cross the donor and target chromosome were transmitted in the expected 1:1 ratio to the progeny (222 and 209, respectively) and no GFP loss was observed, indicating that the rearrangements observed at the target chromosome are induced by the nuclease activity. When female TALEN *trans*-heterozygotes were crossed to wild-type males, we observed a small but significant fraction (1.4%) of RFP-positive flies and a minor fraction (0.3%) of GFP- and RFP-negative flies. Similar crosses with the ZFN SSEs found significantly higher level of activity (ZFN-AAVS1 21.3% and ZFN-AAVS1-Long 11.9% GFP loss), suggesting some nuclease expression and activity in females (Table 1). This observation was unexpected: all donor constructs shared the same testis-specific promoter, which is not active in females (12,13), have an identical 3’-UTR from testis-specific genes, and were inserted at the same integration site, ruling out positional effects of transgene integration or promoter leakage. The presence of cryptic enhancers within the ZFN construct could offer a possible explanation for these observations although a definitive cause has not been yet identified.

In order to assess the stability of the constructs and the nuclease integration events, the activity assay was repeated after a minimum of 10 generations of sibling interbreeding of the donor stock. In this case, *trans*-heterozygous-donor containing flies were back crossed to naive target flies to obtain new donor/target *trans*-heterozygotes in the next generation. For both TALEN and ZFN constructs, we were able to reproduce the initially observed targeting activity (GFP loss) of the SSEs (72% and 83%, respectively, versus the initial 70% and 86%). Next, a fraction of the targeted (homed) males (RFP^+^ and *w*^−^) were individually crossed to new target females in order to determine whether the SSE inserted in the target site was still active and able to perform another round of homing in the following generation. Analysis of the GFP-positive fraction of the offspring indicates if the SSEs were successfully homed (and therefore we would expect cleavage of the GFP locus—resulting in <50% of the progeny being GFP-positive). By contrast, a 1:1 ratio of GFP^+^:GFP^−^ in F2 progeny would indicate an inactive or absent SSE locus. Because the *mini-white* reporter was lost (homed flies are in *w*- background), we could not distinguish inherited donor chromosomes from new homing events; consequently, the phenotypic analysis of GFP expression was the only indicator for scoring SSE activity in the F2. In our TALEN-based SSE, 40% (15/36) of individual crosses resulted in significant (*P* < 0.05, Chi-square) loss of GFP in the F2 progeny (11.9%, Figure 3B). Similarly, 75% (15/20) of individual crosses of the ZFN-AAVS1 expressing SSE lead to the loss of GFP (GFP^+^ fraction: 11%, Figure 3B). The GFP-positive fraction in the crosses in which the SSE appeared to be active is consistent with the rates observed from the original stock (16.8% in the TALEN crosses and 13.8% in the ZFN-AAVS1 crosses). Taken together, these findings suggest that SSE loci are stable during vertical transmission and active when re-exposed to fresh target sites. However, while we found that SSEs could still carry out secondary homing in principle, only 40% and 75% (for the TALEN and ZFN constructs, respectively) of the targeted sites had received functional SSE elements that were able to perform a second round of homing. This indicates that faithful transmission of the SSEs locus during the process of homing is only partially achieved.

**Figure 4.**
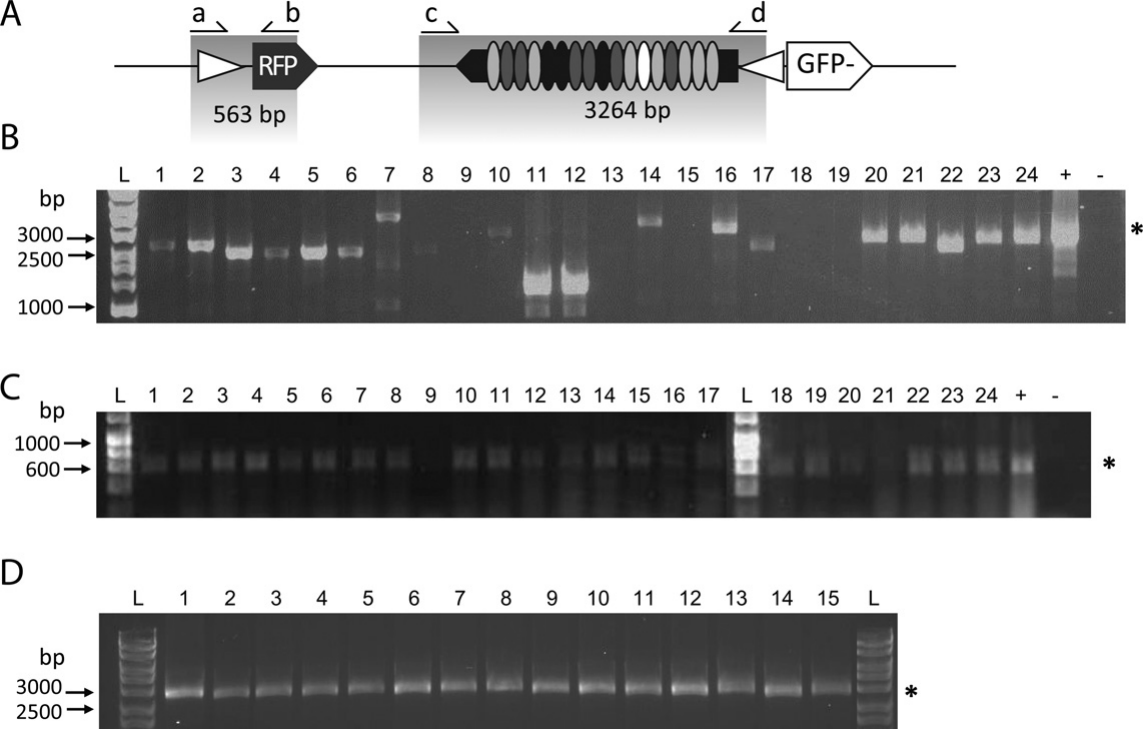
Molecular analysis of F2 dysfunctional homing products. PCR analysis from homed flies that carry dysfunctional TALEN SSE product. (**A**) Schematic of the TALELAT donor construct and the expected size of PCR amplicons (grey areas). (**B**) Primers c–d are expected to generate an amplicon encompassing the TALEN sequence of 3264 bp in size (marked with a star at the right as in the positive control, +). Different sizes of PCR products or failure to amplify were observed from genomic DNA extracted from all non-functional F2 donor-expressing flies. Note the ‘laddering’ effect in the positive control as a PCR artefact of TALEN repeats amplification. (**C**) PCR from all homed flies (except lane 9) gives a positive product of the expected size with primers a–b. (**D**) Primers c–d generate the expected PCR product from TALELAT donor stock flies (3264 bp, star). L: Hyperladder I. + and – indicate positive and negative PCR controls, respectively.

To identify the reasons for the loss of SSE activity, we analysed the F2 RFP^+^ progeny carrying non-functional constructs (i.e. obtained from a homed parent which 50% of the progeny expressed GFP). As shown in Figure 4B, PCR analysis of the sequence carrying the TALEN region yielded amplicons differing from the expected size of SSE or resulted in no amplification at all. By comparison, amplification of the 5’ junction of the donor cassette including the RFP sequence was successful in all but one case (Figure 4C). These observations suggested that when the broken chromosome is repaired, the donor cassette is either incompletely copied into the target site or it is modified in the process. One possible explanation could be direct-repeat recombination between the TALEN modules that exhibit high nucleotide sequence similarity. In addition, events involving the aberrant resolution of HR products between donor and target chromosomes should also be taken into account (6,23). This notion is supported by the observation that no SSE instability occurs when SSEs are directly transmitted from parents to offspring (Figure 4D), but only when, as result of nuclease activity, homing occurs and the SSE is copied between chromosomes. Similarly, when dysfunctional ZFN products were analysed by PCR, we were unable to amplify the region encompassing the ZF modules although we obtained the expected PCR products for fragments spanning both the left and right junction of the donor cassette integration site.

**Figure 5.**
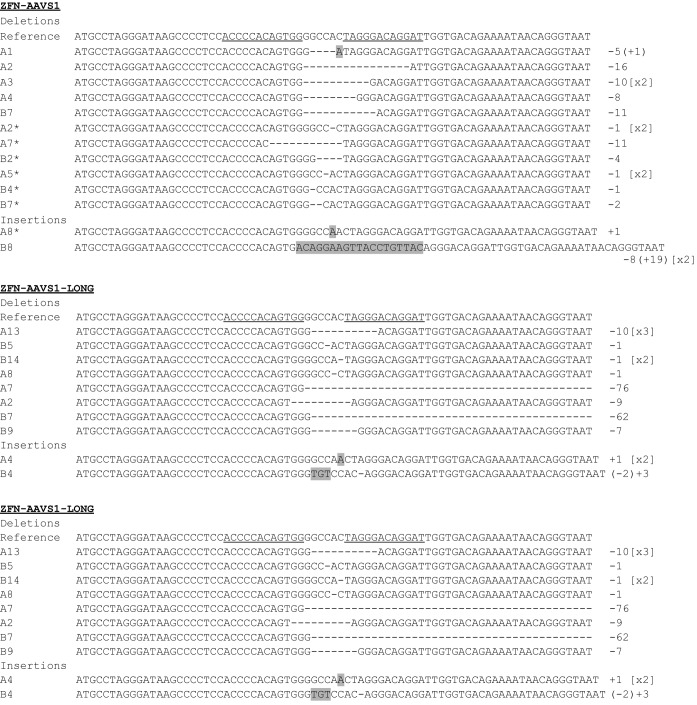
Characterization of NHEJ events originating from TALEN and ZFN activity. Sequencing characterization of imprecise NHEJ events originating from TALEN and ZFN activity, as indicated. The first line shows the GFP coding sequence that includes the nuclease target site (the nucleases binding sequences are underlined). The majority of repair events following ZFN cleavage leave microdeletions in proximity of the cleavage site whereas in the case of TALEN, the repaired chromosome exhibits bigger deletion (up to 300 bp) mainly at the 3’ of the cleavage site. In few cases, partial HR resulted in segmented of donor cassette being inserted in the target site, from either side of the DSB (RFP or *Rcd-1r* sequence). Insertions are highlighted. The numbers of identical repair events are indicated in squared brackets on the right.

To further analyse the activity of the two TALEN and ZFN nucleases, we characterized by PCR and sequencing a number of target chromosomes from GFP and RFP negative progeny for the presence of NHEJ-induced mutations. In agreement with previous studies (24), the ZFN nuclease induced mainly small deletions (from 1–76 bp) and occasionally small insertions (1–19 bp) (Figure 5). TALEN nucleases generated both short (1–50 bp) and large deletions up to 300 bp. In 5 out of 21 events a combination of deletions and substitutions were observed. Interestingly, we also found insertions at the cleaved locus that appear to have resulted from partially resolved recombination, using either side of the donor cassette of the homologous chromosome as template. In 3 out of 21 sequenced events, we observed insertion of up to 400 bp of the RFP ORF, and in 1 case insertion of 173 bp of the *Rcd-1r* promoter within the GFP sequence, consistent with the previous observation showing that a fraction of homing events lead to dysfunctional SSE products.

### Genomic integration of artificial nucleases does not affect fly fitness

ZFN and TALEN nucleases have been widely used to modify a number of target genes in transient expression experiments via either direct mRNA injection or expression vectors. However, there is little information on the effect of stable germline transformation and endogenous expression of these nucleases in animal genomes. We therefore looked for signs of toxicity due to non-specific or off-target activity of these nucleases in our model system. No obvious fitness defects in terms of general stock viability were observed in endonuclease expressing flies. After at least 10 generations the oviposition and egg hatching rates of ZFN and TALEN expressing stocks did not differ significantly from the wild-type control (Supplementary Figure S1). In order to address if genetic modifications had been introduced at off-target loci as a result of TALELAT and ZFN-AAVS1 activity, we identified the genomic locations that showed the highest similarity to their exogenous target site and characterized them at the molecular level. The analysis of seven different motifs closely resembling the AAVS1 site in the *Drosophila* genome (5 bp mismatches, see Supplementary Table S1) showed no obvious evidence of cleavage in flies expressing ZFN-AAVS1. Similarly, genomic loci that most closely resemble the TALELAT target sequence (14–16 bp mismatches) were found to be unaltered in the TALEN expressing flies. These observations suggest high specificity and low toxicity of these nucleases at least at detectable frequencies.

### SSE ability to invade target populations

**Figure 6.**
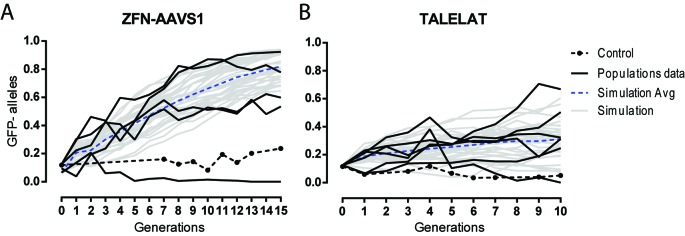
Population invasion experiments of ZFN and TALEN SSEs. Fraction of GFP-negative alleles in populations of flies monitored for up to 15 generations after a single initial release of 20–25% of SSE *trans-*heterozygous males in a GFP target population. Each data set refers to individual populations of 400 and 100 flies each for (**A**) ZFN-AAVS1 and (**B**) TALEN, respectively. The experimental points from (A) five and (B) six populations (black lines) were compared to 30 independent iterations of a stochastic model (grey line) based on the experimental data of cleavage and homing reported in Table 1. The average of the 30 stochastic simulations is also plotted (dotted blue lines). As a control *trans*-heterozygous males were introduced into a population of 400 refractory (non-cleavable) target flies at an initial frequency of 25% (black dotted line).

Despite the high cleavage and homing rates observed for SSEs in our assays, the fraction of dysfunctional products generated could affect the ability of the SSE to invade a target population. To test the invasiveness of these SSEs, we introduced SSE-bearing *trans*-heterozygous males into a population of flies homozygous for the GFP^+^ target chromosome, and monitored the fraction of GFP alleles in the population for up to 15 consecutive generations (Figure 6). Experimental data for populations of 400 and 100 total individuals for ZFN and TALEN SSEs release, respectively, were compared with the output of stochastic models based on the experimental parameters of cleavage, homing and NHEJ rates reported in Table 1, and assuming no fitness difference between genotypes. As a control, we used a refractory (non-cleavable) target population initially obtained from an individual in which SSE-induced NHEJ repair had destroyed the target site. Introduction rates of *trans*-heterozygous flies were 15–25% (see Materials and Methods for details), each individual carrying both a GFP^+^ and a GFP^−^ (donor) allele: therefore the initial fractions of GFP^-^ alleles in the populations ranged from 7.5 to 12.5%. In four out of five populations, the ZFN-based SSE could convert 53–92% of the GFP alleles in 15 generations, in agreement with the predicted stochastic model (Figure 6A). In the population in which the initial GFP-negative allele frequency was 7.5%, the SSE bearing flies were lost from the population after five generations, after which almost all individuals were expressing functional GFP alleles. When ZFN-SSEs were released in the control population of refractory target flies, the fraction of nuclease alleles was maintained around the initial frequency, indicating that the spread of the ZFN-SSE in the target populations are the result of the SSE activity.

When the TALEN-based SEEs were released in the target populations, we observed high variability between the different populations, as predicted by our stochastic model (Figure 6B). The GFP-negative fraction in five out of six populations ranged from 25 to 67% after 10 generations; in one population, the nuclease expressing donor alleles were eliminated while the donor flies released in the control population oscillated around the initial frequency. The generation of dysfunctional products at high frequency for any given homing event is likely to contribute to the observed (and predicted) variability.

## DISCUSSION

Taken together our results provide a proof of principle that both TALEN and ZFN nucleases can be adapted to generate SSEs that can spread into the genome of naive host populations with modalities similar to those described for HEGs. Successful HR events induced by SSE activity reconstitute the molecular hallmarks of homing. The synthetic ZFN- and TALEN-based SSEs induced overall transmission in the *Drosophila* germline at a level higher than the homing rate observed for a natural homing endonuclease, *I-Sce*I, expressed under the same regulatory sequences (13). This is not obvious, since site-specific cleavage activity is insufficient by itself to ensure efficient homing (6) and HR propensity for resolving DBS depends on the target/nuclease combination—i.e. not solely on the nuclease cleavage efficiency. Unlike HEGs, a high rate of NHEJ was also observed, a finding that is potentially linked to the fact that ZFNs and TALENs generate 5’-overhangs as opposed to the 3’-overhangs generated by most HEGs (25). A similar observation has been made by Kuhar and colleagues, which reported a marked difference in repair pathway used after a TALEN-induced break compared to *I-Sce*I-induced break: the former favouring gene disruption (NHEJ) and the latter HR-dependent gene conversion (25). Whether the difference in repair pathway is due to the difference in overhang polarity or other biochemical properties of the nucleases remains unclear.

TALENs are now a standard tool for genome editing and have quickly replaced ZFNs, given their higher versatility which allows the targeting of a wider array of sequences and for the ability to easily assemble custom-made TALENs without the need for commercial synthesis (26–28). TALENs are usually 1.5–2 kb larger than ZFN nucleases and this size difference may have a detrimental impact on homing activity, however we observe no correlation between donor construct size and homing activity. We found that the ZFN-AAVS1-Long construct, which is of similar size to a donor cassette hosting a full pair of TALENs, induced homing at the same rate as the shorter ZFN-AAVS1 containing construct.

Genomic integration of ZFN and TALEN nucleases could affect organismal fitness due to off-target activities of the enzymes. We showed that viability, fecundity and fertility were not measurably affected in our fly stocks expressing the nucleases. The activity of the nucleases after several generations of sibling interbreeding when exposed to fresh target sites was comparable to the original stock, indicating that integration and vertical transmission of the nucleases is stable. We have not performed an exhaustive investigation of potential off-target effects on the whole *Drosophila* genome but molecular analysis of putative off-target sites did not uncover any evidence of off-target cleavage, at least at frequencies higher than 12.5%.

In addition, and in contrast to HEGs, which have been evolutionary selected for their high homing efficiency, recombinational events associated with TALEN- and ZFN-induced break repair generate non-functional products at higher rate than HEGs. This limitation of TALEN is likely related to the highly repetitive nature of their repeat-variable diresidue (RVD) modules. While this does not prevent the generation of stably integrated TALEN SSEs or, as we have shown, their vertical inheritance, it limits their faithful transmission via homing. Similar studies using natural or engineered HEGs showed that the fraction of dysfunctional products as result of partial homing ranges from 0 to 11% (6,14). We observed that about 60% and 25% of homing events involving TALEN- and ZFN-based SSE, respectively, lead to products which are not able to perform a second round of homing, probably due to intra-modular recombination events or mispairing of the long, tandem RVD repeats during DSB repair. We partly alleviated this problem by expressing a monomeric TALEN that recognizes a palindromic target sequence, thus halving the number of repeats. Monomeric TALENs (or compact TALEN) have recently been developed as a fully functional nucleases with high activity and specificity *in vivo* (29), opening the possibility for more compact TALEN design. However, the full exploitation of modular nuclease for generating SSEs will require sequence engineering to reduce the repetitive DNA content of individual modules. Such approaches could be applied to stabilize TALEN intra-molecule repeats as well as ZFN inter-molecule repeats. In addition, ZFN-TALEN hybrid nucleases (30) could also be exploited to reduce the repetitive complexity of the system while retaining the sequence specificity and flexibility of the modular architecture.

We have shown that off-the-shelf SSEs could potentially invade a host population but the accumulation of dysfunctional homing products limits their efficiency, especially for TALEN-based systems that do exhibit a high homing rate. As a result, TALEN-SSEs, in our model system, were unable to invade a target population. In addition, the homing derivatives with deletions in the TALEN modules may generate less specific proteins that cleave secondary targets at lower frequency, jeopardizing the site-specificity of the system. ZFNs efficiently target wild-type alleles, but their ability to invade populations is limited by their relative lower homing rate. Consequently, the practical application of SSEs at the population level will require a substantial improvement in their genetic stability.

Our findings provide important information for elucidating the mode of action of endonuclease-based selfish elements. In addition, the development of SSEs that combine the engineering flexibility of TALENs and ZFNs has diverse implications, including control of disease vectors, for example, facilitating the replacement of wild-type insect populations with variants that do not transmit diseases.

## SUPPLEMENTARY DATA

Supplementary Data are available at NAR Online.

SUPPLEMENTARY DATA
