# Development of synthetic selfish elements based on modular nucleases in Drosophila melanogaster

**DOI:** 10.1093/nar/gkv117

**Published:** 2015-02-11

**Authors:** A. Simoni, C. Siniscalchi, Y.S. Chan, D.S. Huen, S. Russell, N. Windbichler, A. Crisanti

Nucleic Acids Res. 2014 Jun;42(11):7461–72. doi: 10.1093/nar/gku387.

The authors have accidently duplicated the ZFN-AAVS1-LONG panel in Figure 5 of the above article. A new figure is provided below.

**Figure 5.**
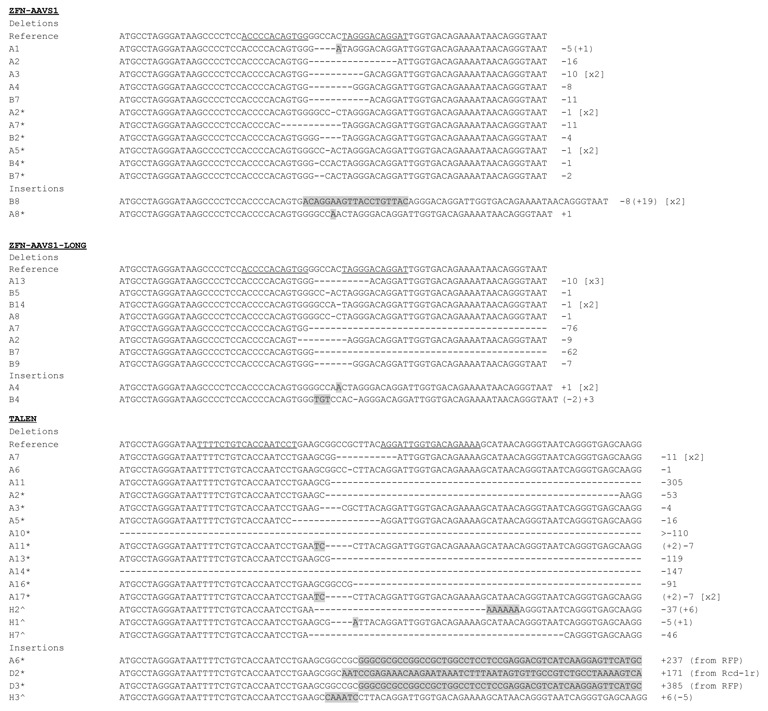
Sequencing characterization of imprecise NHEJ events originating from TALEN and ZFN activity, as indicated. The first line shows the GFP coding sequence that includes the nuclease target site (the nucleases binding sequences are underlined). The majority of repair events following ZFN cleavage leave microdeletions in proximity of the cleavage site whereas in the case of TALEN, the repaired chromosome exhibits bigger deletion (up to 300 bp) mainly at the 3’ of the cleavage site. In few cases, partial homologous recombination resulted in segmented of donor cassette being inserted in the target site, from either side of the DSB (RFP or Rcd-1r sequence). Insertions are highlighted. The numbers of identical repair events are indicated in squared brackets on the right.

This error does not affect the results or conclusion of the article.

The authors wish to apologise to readers for the inconvenience caused.

