# Supplementary material for: Development of synthetic selfish elements based on modular nucleases in Drosophila melanogaster
Source: Nucleic Acids Res. 2014 May 6;42(11):7461–72. doi: 10.1093/nar/gku387 (PMC4066794; doi:10.1093/nar/gku387)
Supplement: SUPPLEMENTARY DATA [file supp_42_11_7461__index.html]

SUPPLEMENTARY DATA 

# Development of synthetic selfish elements based on modular nucleases in *Drosophila melanogaster*

## SUPPLEMENTARY DATA

**Files in this Data Supplement:**

- Supplementary Data
