# Supplementary material for: Development of synthetic selfish elements based on modular nucleases in Drosophila melanogaster
Source: Nucleic Acids Res. 2014 May 6;42(11):7461–72. doi: 10.1093/nar/gku387 (PMC4066794; doi:10.1093/nar/gku387)
Supplement: SUPPLEMENTARY DATA [file supp_gku387_nar-00045-f-2014-File008.pdf]

Supplementary information

Development of Synthetic Selfish Elements Based on Modular Nucleases in *Drosophila melanogaster*

Alekos Simoni[1], Carla Siniscalchi[1], Yuk-Sang Chan[2], David S. Huen[2,3], Steven Russell[2], Nikolai Windbichler[1], Andrea Crisanti[1]

[1] Department of Life Sciences, Imperial College London, South Kensington Campus, London SW7 2AZ, United Kingdom

[2] Department of Genetics, University of Cambridge, Downing Street, Cambridge CB2 3EH, United Kingdom

[3] Current address: Department of Applied Sciences, University of Wolverhampton, Wolverhampton WV1 1LY, United Kingdom.

Correspondence should be addressed to A.C.: acrs@imperial.ac.uk

A

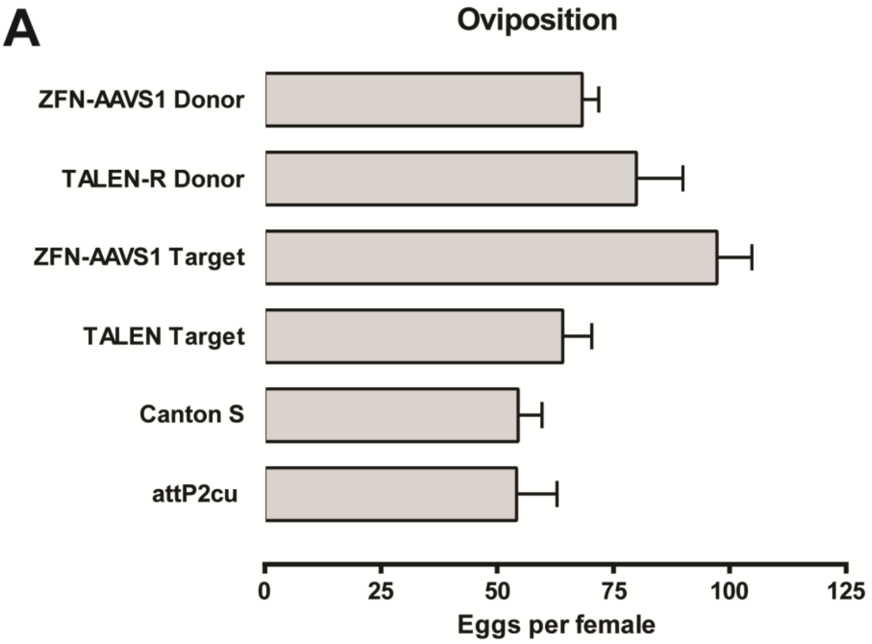

B

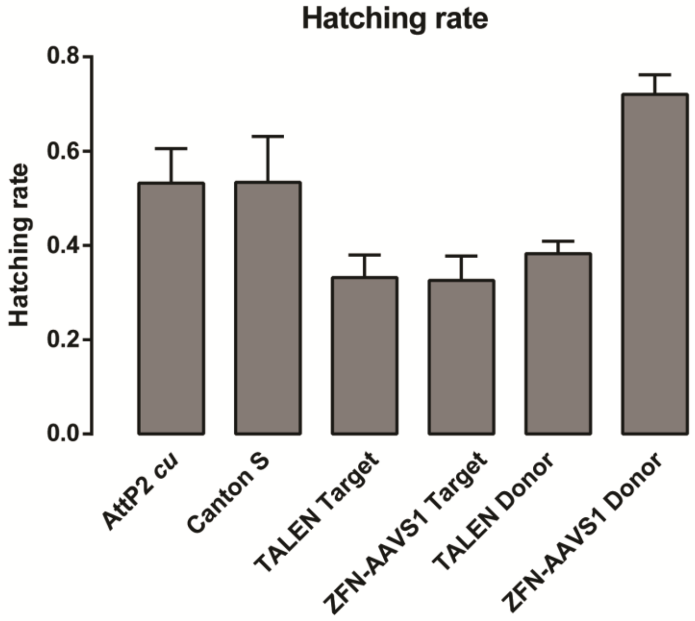

Supplementary Figure 1: Fitness analysis of donor and target lines: **A.** Oviposition analysis: Two to three females were let to lay eggs in a vial for 4 consecutive days and the total number of eggs laid per female per day is shown. At least 4 replicas were performed and SEM is plotted. No statistical difference was found compared to *attP*2 control (1way ANOVA with Tukey's post-test). **B.** Hatching rate analysis: The bars show the ratio values between the total number of eggs laid and the total number of adults hatched. Test performed with 1way ANOVA analysis did not find significant difference amongst the lines. Three to six replicates per line were performed. Error bars indicate SEM between independent experiments.

<table>
<tr><th>Alignment</th><th>Configuration</th><th>Mismatch</th><th>Position</th></tr>
<tr><td colspan="4">AAVS1</td></tr>
<tr><td>Target: 30 ATCCTGTCCCTANNNNNNCCACTGTGGGGT 1
|||| | || | ||||||||| ||
Ref: 10670442 ATCCCGCACCGATCTATTCCACTGTGGTGT 10670471</td><td>L-6-R</td><td>5</td><td>2R</td></tr>
<tr><td>Target: 29 ATCCTGTCCCTANNNNNCCACTGTGGGGT 1
||||||| | | | |||||| |||
Ref: 20771575 ATCCTGTACATCTGAAGCTACTGTGCGGT 20771603</td><td>L-5-R</td><td>5</td><td>3R</td></tr>
<tr><td>Target: 1 ACCCCACAGTGGNNNNNTAGGGACAGGAT 29
|| ||| ||| |||||||||||
Ref: 2317114 ACACCAAGCTGGCCAATCAGGGACAGGAT 2317142</td><td>L-5-R</td><td>5</td><td>3R</td></tr>
<tr><td>Target: 1 ACCCCACAGTGGNNNNNNTAGGGACAGGAT 30
||||||| |||| | | |||| ||
Ref: 5300848 ACCCCACTGTGGCTCCAGAAAGAACAGAAT 5300877</td><td>L-6-R</td><td>5</td><td>X</td></tr>
<tr><td>Target: 1 ACCCCACAGTGGNNNNNCCACTGTGGGGT 29
|||||| ||||| ||| || |||
Ref: 14763780 ACCCCAAAGTGGACGCTCCATTGCGGGTG 14763808</td><td>L-5-L</td><td>5</td><td>2R</td></tr>
<tr><td>Target: 1 ATCCTGTCCCTANNNNNTAGGGACAGGAT 29
||||| |||||| |||| ||| |
Ref: 531280 ATCCTATCCCTATTCCATAGGTTGAGGGT 531308</td><td>R-5-R</td><td>5</td><td>3R</td></tr>
<tr><td>Target: 1 ACCCCACAGTGGNNNNNCCACTGTGGGGT 29
|||||| | || ||||| ||| ||
Ref: 19251423 GCCCCACTGGGGTAACACCACTTTGGTGT 19251451</td><td>L-5-L</td><td>5</td><td>3L</td></tr>
<tr><td colspan="4">TALELAT</td></tr>
<tr><td>Target: 1 TTTTCTGTCACCAATCCTNNNNNNNNNNNNNNAGGATTGGTGACAGAAAA 50
|||||||| | |||||| | | | | | |||||||
Ref: 11654491 TTTTCTGTTATAAATCCTTTCAATTGCTAGCTTGCAATTGGGTCAGAAAA 11654540</td><td>R-14-R</td><td>9</td><td>3L</td></tr>
<tr><td>Target: 1 TTTTCTGTCACCAATCCTNNNNNNNNNNNNNNNNAGGATTGGTGACAGAAAA 52
| |||||||||| ||||||| | | ||||
Ref: 19986190 GCGTGCATCACCAATCCGCTGGCCGAGCTAGTTGAGGATTGCTTGCGCAAAA 19986241</td><td>R-16-R</td><td>12</td><td>3R</td></tr>
</table>

Supplementary Table 1: Alignment of putative off-target sites in the *Drosophila* genome with ZFN and TALEN recognition sequences. Alignment was performed considering ZFN left-right (L-R), left-left (L-L) or right-right (R-R) configuration (as indicated) with 5 to 6 bp as spacer (N). For TALELAT we search for palindromic sites with spacer length of 14 to 25 bp. The number of mismatches between the putative off-target and target sequence is indicated. The chromosomal location of the putative off-target site is shown.
